# Elovanoids downregulate SARS-CoV-2 cell-entry, canonical mediators and enhance protective signaling in human alveolar cells

**DOI:** 10.1038/s41598-021-91794-z

**Published:** 2021-06-10

**Authors:** Jorgelina M. Calandria, Surjyadipta Bhattacharjee, Nicholas J. Maness, Marie-Audrey I. Kautzmann, Aram Asatryan, William C. Gordon, Khanh V. Do, Bokkyoo Jun, Pranab K. Mukherjee, Nicos A. Petasis, Nicolas G. Bazan

**Affiliations:** 1 Neuroscience Center of Excellence, School of Medicine, Louisiana State University Health New Orleans, 2020 Gravier Street, Suite D, New Orleans, LA 70112 USA; 2 Tulane National Primate Research Center, Covington, LA USA; 3 Department of Microbiology and Immunology, Tulane University School of Medicine, New Orleans, LA USA; 4 Department of Chemistry and Loker Hydrocarbon Research Institute, University of Southern California, Los Angeles, CA USA

**Keywords:** SARS-CoV-2, SARS virus

## Abstract

The pro-homeostatic lipid mediators elovanoids (ELVs) attenuate cell binding and entrance of the SARS-CoV-2 receptor-binding domain (RBD) as well as of the SARS-CoV-2 virus in human primary alveoli cells in culture. We uncovered that very-long-chain polyunsaturated fatty acid precursors (VLC-PUFA, n-3) activate ELV biosynthesis in lung cells. Both ELVs and their precursors reduce the binding to RBD. ELVs downregulate angiotensin-converting enzyme 2 (ACE2) and enhance the expression of a set of protective proteins hindering cell surface virus binding and upregulating defensive proteins against lung damage. In addition, ELVs and their precursors decreased the signal of spike (S) protein found in SARS-CoV-2 infected cells, suggesting that the lipids curb viral infection. These findings open avenues for potential preventive and disease-modifiable therapeutic approaches for COVID-19.

## Introduction

The high transmissibility of Severe Acute Respiratory Syndrome-coronavirus 2 (SARS-CoV-2) is due, at least in part, to infectivity in lung type II alveolar cells^1^ SARS-CoV-2 triggers a wide range of disease phenotypes with severe acute respiratory distress syndrome (ARDS), including interstitial pneumonia^2^ and viral sepsis^3^. Here, we tested if the pro-homeostatic lipid mediators, the elovanoids (ELVs)^4–7^, would block the entrance of the spike (S) protein receptor-binding domain (RBD) that would prompt a protective response against SARS-CoV-2 infection.

**Figure 1:**
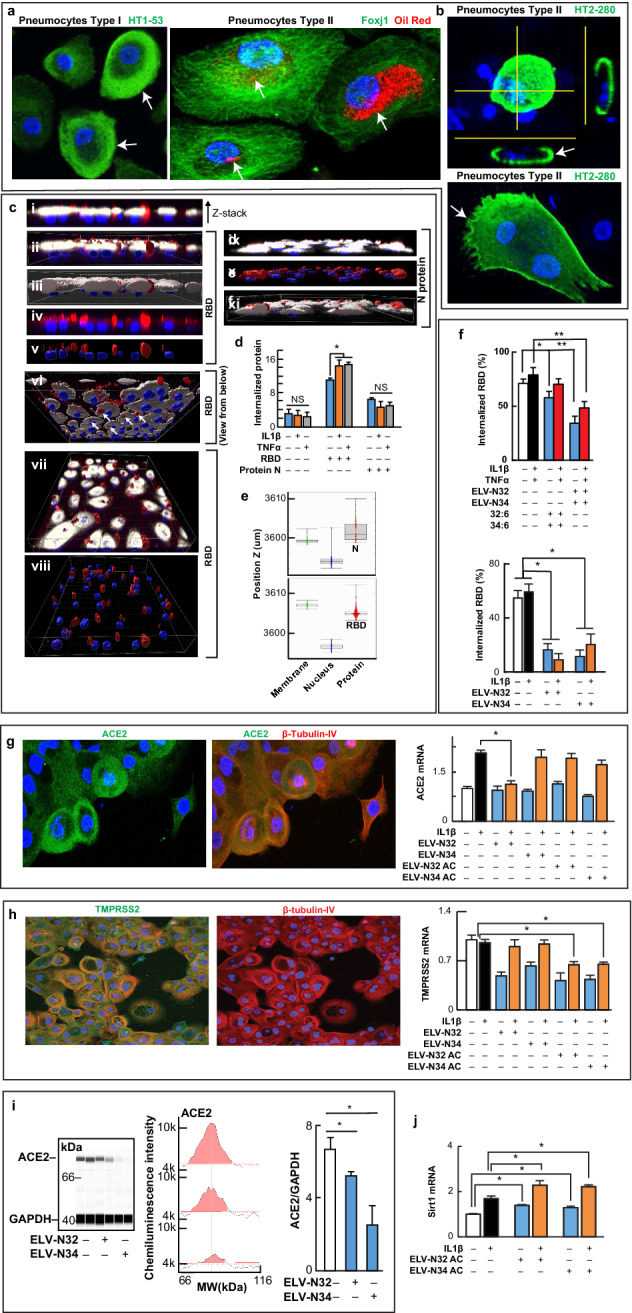
Specific internalization of Spike’s protein RBD is reduced by Elovanoids and their precursors in human primary alveolar cells. (**a**) Characterization of Pneumocytes type I (left panel) and type II (right panel) in the alveolar culture. Pneumocytes type I are reactive to HT1-53 (green), and pneumocytes type II are positive for oil red (red), a marker for the type II lipid/lamellar bodies and for ciliated cell marker Foxj1 (green) which is required for cilia formation and is an early marker of epithelial cell differentiation, recovery, and function. (**b**) Pneumocytes type II stained positive to HT2-280 labeled (green) type II human lung cells. (**c–e**) Differential entrance of S protein vs. N in human alveolar cells in culture. Z-stack shows signal distribution (upper panel), and in mobile cells undergoing cell division (lower panel). (**c**) XZ planes of a Z-stack showing white: membrane, red: RBD tagged with Alexa Fluor 594 and blue nuclei. i through v depict S protein view from XZ plane digitalized of the IMARIS image; vi to viii view from below, above and digitalized showing the internalized protein (white arrows); ix to xi XZ plane depicting the labeled N protein that remains in the surface or within the plasma membrane. Viral Nucleocapsid N protein was tagged with Alexa Fluor-594. XZ plane of a Z-stack image of alveoli culture after 24 h exposure to N protein. (**d**) Quantification of internalized signal when no protein, N and S in the presence or absence of IL1β or TNFα. (**e**) position of the protein signal in the Z-axes. The plots show digitalized elements position in the Z-axis from 2 representative images of N protein (top panel), and for RBD (bottom panel). The membrane thickness was taken as reference in each picture to determine % of proteins above (free protein), within (bound protein), and below (internalized protein) where green dots are membrane signal, blue dots are nuclei, and red dots are protein. The Y-axis denotes the μm thickness of the image or Z. The boxes showed the median (middle), and the upper and lower lines depict the Quartile 1 and 3. The whiskers showed the maximum and minimum position adopted in the Z-axes. (**f**) RBD protein internalization decreased by ELV-N32, 34, and their precursors, VLC-PUFAs 32:6 and 34:6. The addition of ELV-N32 and ELV-N34, 2 μM of the acetylene form together (top panel) or 1 μM separated methyl ester form (bottom panel), and their precursors 32:6 and 34:6 (2 μM) showed a decrease in the Alexa-Fluor 594-RBD internalization. (**g–i**) Expression of ACE2 and TMPRSS2 in alveolar cells. Immunocytochemistry of alveolar cells shows signal of ACE2 (**g,** left panels) and TMPRSS2 (**h,** left panels) in pneumocytes type II expressing β-tubulin-IV and the mRNA expression for both genes by Taqman qPCR (**g–h**). (**i**) ACE2 protein quantification (by Jess-capillary Wester Blots-Protein Simple: left panel depicts ACE2 bands at 90 KDa for ACE2 and 40KDa for GAPDH; in the middle panel is shown the area below the pic intensity used to quantified the ACE2 band and the plot shows the quantitation of N = 2 samples of alveolar cells + /− ELV-N32 and N34. (**j**) The expression of Sirt1 mRNA, an ELV-N32 and N34 target. Bars depict the mean of N = 24 pictures plus/minus standard error. *P* < 0.05. Membrane (white), S or N Protein (red), and nuclei (blue). Boxes color code all the histograms in the figure.

Lung alveoli viral attachment through the S protein RBD to ACE2 is followed by proteolytic activation for fusion and viral cell entry^8–10^. We use human alveolar primary cell cultures (Fig. 1a, and Extended Data Fig. 1). Most of the cells are type II (oil red, specific marker, Fig. 1a, right panel, and Extended Data Fig. 1, bottom panels) and positive to Foxj1, HT2-280 antigen, and β-tubulin IV (Fig. 1a, right panel; Fig. 1g,h and Extended Data Fig. 1). Pneumocytes type II are also mobile, showing lamellae or filopodia positive to HT2-280, a specific type II (Fig. 1b). We exposed these cells to RBD (from S protein)-Alexa 594 for 24 h. In parallel, we used Nucleocapsid protein N as a specificity control of RBD internalization. In 3D reconstructions of Z-stack images (Imaris software, Bitplane, UK), the lipophilic staining (cell mask) shows a dense membrane above the nuclear zone that is localized close to oil red (Fig. 1a,c i–viii). A below view (Fig. 1c vi) shows that the RBD protein signal (red) passes through the membrane (white) to the intracellular space surrounding the nucleus (blue), and can also be seen in the above view (Fig. 1c vii,viii). Herein, we demonstrate for the first time that RBD was shown to be internalized in SARS-CoV-2 since previous work have shown the same for SARS-CoV-1^11^. When IL1β or TNFα was added, the internalized RBD signal was increased (Fig. 1d). In addition, Nucleocapsid (N) protein, a structural viral protein not involved in ACE2 and SARS-CoV-2 interaction^12^, is at the same level as the control with no protein added that accounted for autofluorescence. RBD was internalized at higher rates than N. In digitalized images, plotted vs. Z-axis in a Z-stack shows the differential position of the N protein versus the RBD with respect to the membrane level (Fig. 1c i–v,ix–xi,d). This observation documents that the N protein remains on the membrane and the extracellular side while the RBD spans intracellularly, passing through to the cytoplasm and demonstrating that RBD internalization is specific and dependent on IL1β and TNFα (Fig. 1d,e). We used IL1β and TNFα to mimic inflammatory injury in the lung cell to determine if that would facilitate the entrance of the RBD peptide, and as ELVs are characterized as pro-homeostatic lipids, we hypothesized that ELVs would block the proinflammatory effect on RBD entrance. RBD-Alexa 594 internalization was decreased + /− IL1β + TNFα when ELV-N32 and ELV-N34 were added (Fig. 1f, upper panel). In addition, acetylenic ELV-N32 or ELV-N34 (Extended Data Fig. 2) showed a steep decrease in RBD protein internalization + /− IL1β (Fig. 1f, lower panel). Moreover, the addition of the ELVs precursors 32:6 or 34:6 reduces RBD located below the membrane, suggesting that the pneumocytes convert these precursors into ELVs and thus prevent RBD internalization (Fig. 1f, upper panel).

The reduction in RBD internalization is partially due to a decrease in ACE2 since acetylenic ELV-N32 or ELV-N34 decreases ACE2 mRNA and methyl-ester elovanoids also reduce the protein content in pneumocytes type II (Fig. 1g, plot, and Fig. 1i). In addition, ELV-N32 decreased TMPRSS2 expression (Fig. 1h) in the presence of IL1β (Fig. 1h, plot).

Since ELVs stimulate protective protein expression in cells confronted with uncompensated oxidative stress^5–7^, we next explored if these lipids under conditions that downregulate canonical SARS-CoV-2 cell-entry mediators in pneumocytes will also activate protective proteins synthesis. We found that ELV heightens the expression of Sirtuin 1 (Fig. 1j), RNF146 (Extended Data Fig. 3a,b), PHB, Bcl-Xl, and Bcl2 (Extended Data Fig. 3c–e). These proteins are involved in pro-homeostatic cellular functions. Sirtuin 1 (Silent information regulator factor 2-related enzyme 1) is a NAD(+)-dependent deacetylase of histone and non-histone proteins and transcription factors, and its regulatory functions target inflammation, aging, mitochondrial biogenesis, and cellular senescence^13^. RNF146 is an E3 ubiquitin-protein ligase that degrades parsylated proteins, thus protecting cells from Parthanatos cell death^14^. PHB (prohibitin type I) functions comprise scaffolding mitochondrial protein, adaptor in membrane signaling, transcriptional co-regulator, and neuroprotection^6^. Bcl-XL and Bcl2 downregulate apoptosis and inflammasome formation^15^. Our data suggest that, in addition to halting the entrance of the RBD, ELVs in the lung curb cell-damaging/apoptotic events and thus sustains homeostasis by counteracting inflammation over-activation by the formation of protective proteins.

**Figure 2:**
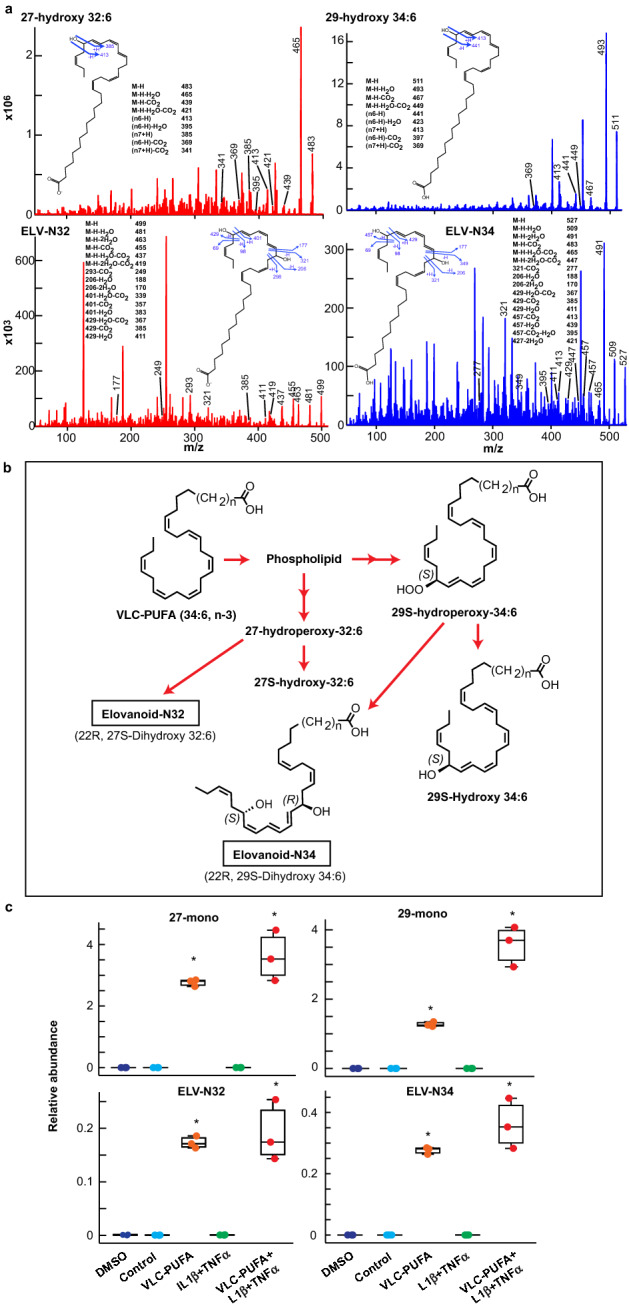
Fate of VLC-PUFAs (C32:6 and C34:6, 2 μM each) added to human primary alveolar cells cultures towards ELV synthesis. (**a**) 27-monohydroxy-32:6, 29-monohydroxy-34:6, and ELVs are formed. Full fragmentation of 27-monohydroxy-32:6 and 29-monohydroxy-34:6 show matches to their theoretical peaks (top panels). The inserts depict the structures of 27-monohydroxy-32:6 and 29-monohydroxy-34:6 along with the product ions as they are cleaved at a given bond. The complete structures of ELV-N32 and ELV-N34 were confirmed to be: ELV-N32: (14*Z*,17*Z*,20*R*,21*E*,23*E*,25*Z*,27*S*,29*Z*)-20,27-dihydroxydo-triaconta-14,17,21,23,25,29-hexaenoic acid; ELV-N34: (16*Z*,19*Z*,22*R*,23*E*,25*E*,27*Z*,29*S*,31*Z*)-22,29-dihydroxytetra-triaconta-16,19,23,25,27, 31-hexaenoic acid (bottom panels). (**b**) Synthesis Pathways for ELV-N32 and ELV-N34 from VLC-PUFAs 34:6, n-3**. (c**) Relative abundance of the elovanoids N32 and N34 (bottom panels) and their intermediates 27 mono-hydroxy and 29 mon-hydroxy (top panels) when the alveolar cells are exposed to the precursors VLC-PUFAs: 32:6 and 34:6 in the presence or absence of 10 ng/ml IL1β and 10 ng/ml TNFα for 24 h. The plot shows the box upper limit 3rd Quartile, bottom side 1st quartile, middle line: the median and the whiskers denote the maximum and minimum observations. *P < 0.05 in t-test comparisons with the respective control.

An evolving question prompted by our data is whether alveolar cells in culture can synthesize ELVs. Thus, we incubated human alveolar cells with the precursors VLC-PUFAs (32:6 or 34:6) and then analyzed the products by LC–MS/MS. Interestingly, we found that ELVs are in fact, formed. ELV-N32 was synthesized where the precursor 32:6 was added and not in cells exposed to 34:6. Inversely, ELV-N34 was found in the cultures were 34:6 was added and not in cells exposed to 32:6 (Fig. 2a,c). These results demonstrate that alveolar cells are endowed with pathways for the biosynthesis of ELV-N32 and ELV-N34 (Fig. 2b). We show MS fragmentation for stable derivatives of intermediaries (Fig. 2a–c) as well as of ELVs themselves (Fig. 2a). Moreover, we uncovered that ELVs were actively released from cells to the incubation media, indicating that they act both as autocrine and paracrine mediators. The addition of ELVs and their precursors to the human lung cells in culture for extended periods of time (Extended Data Video 1) did not affect cell survival, in agreement with the fact that the cell possesses the molecular machinery to synthesize them, and thus they are also likely able to degrade them.

Our findings contribute to broadening our understanding of the duality of ACE2 in lung function and diseases. In health, ACE2 fosters lung homeostasis by generating Ang-(1–7) and enhancing host defense that would counteract ACE2 virus-induced downregulation of proinflammatory signaling. Herein, we show that ELVs uncover another participant when RBD of the S protein binds to ACE2 and enters alveolar cells in culture. The ELVs are likely part of a fast and coordinated pro-homeostatic inflammatory downregulatory response. To be tested in the future is the prediction that delayed ELV-mediated protective responses would lead to severe lung and systemic inflammation. So direct virus-triggered cell damage is critical, but also the activation of the induction of protective proteins. Is diet engaged in building precursors of ELVs in the lung? Diet has been shown to affect ACE2 expression^16^ and the supply to build ELV precursors^7,17^. This may contribute to explaining why some patients develop hyper-inflammatory/immune responses and severe disease, but others experience mild or even asymptomatic COVID-19. Questions that remain to be addressed include whether the expression of the protective proteins identified here in the alveoli are activated all at once? Are they coordinated with adaptive immune responses to limit virus spread? Are enzymes for ELV synthesis under tight transcriptional control so that the mediators are expressed at appropriate times and/or levels? To our knowledge, ELVs are the first protective mediators to be identified in the human alveoli confronted with the RBD of the S protein.

**Figure 3:**
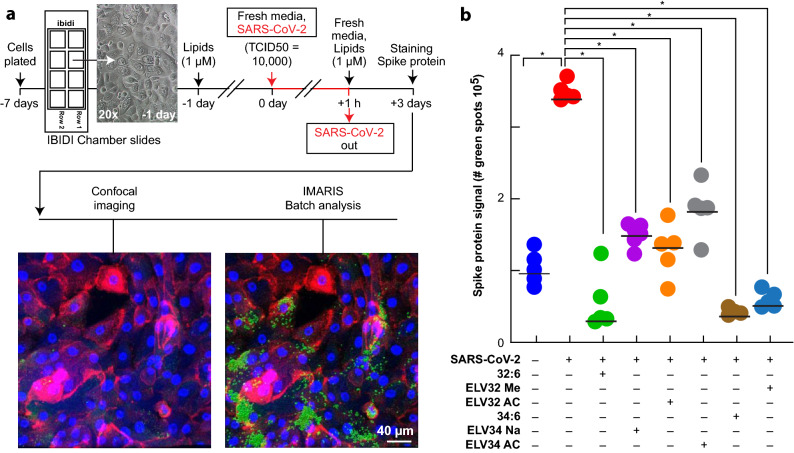
Addition of VLC-PUFAs (C32:6 and C34:6, 1 μM) or ELV-N32 or ELV-N34 decreased viral infection in human primary alveolar cells. (**a**) Timeline of the treatment and infection of human alveolar cells in culture. The cells were plated and let them seat for 7 days until confluency. The day before infection, cells were pre-incubated with 1 μM of Elovanoid 32 methyl-ester (Elv32 Me), Elovanoid 34 sodium salt (Elv34 Na), Elovanoid 32 Acetylenic (Elv32 AC), Elovanoid 34 Acetylenic (ELV34AC) or their precursors, the very long fatty 32:6 or 34:6. On the day of the infection, the medium was removed, the virus was added in a small volume for an hour and then replaced by fresh medium containing the lipids mentioned before. After 3 days, the cells were fixed and stained, and confocal images were taken. Imaris unbiased Batch analysis was performed. The cells were counterstained for membrane (red) and nucleus (blue) with cell mask and Hoechst. (**b**) The number of spots of green signal corresponding to Spike protein was counted and plotted. The data were plotted and analyzed with Graph pad. Normality and Anova were tested. *P < 0.05 in t-test comparisons with the respective control.

To determine the effect of the lipids in viral infection to the alveolar cells, we exposed them to the SARS-CoV-2 to record impedance at confluence, taking images every hour for visual confirmation. We observed no changes in impedance when comparing no-virus exposure with two different Median Tissue Culture Infectious Dose (TCID50), 1000 and 10,000. The time/image composites (depicted in Extended Data Video 2) show that as soon as infected cells are lifted, the neighboring cells fill the gap resulting in no difference in impedance. In light of this observation, we infected human alveolar cells with SARS-CoV-2 for 3 days and immunostained them using anti-Spike protein (Fig. 3a). The infection resulted in an intracellular Spike protein signal (Fig. 3b). The addition of ELV-N32 methyl-ester, ELV-N34 sodium salt, and both acetylenic ELVs and their precursors, VLC-PUFAs 32:6 and 34:6, remarkably decreased the signal of S protein in the cells, suggesting that the lipids prevented the viral infection of the alveolar cells. The VLC-PUFAs may exert this protective effect by the synthesis of ELVs or by modifying the cell surface membrane lipidome remodeling that would disrupt tetraspanin-membrane microdomains^18,19^ to contribute to blocking SARS-CoV-2 virus cell binding and entrance, and in addition, perturb endosome formation hindering virus replication.

Additional research will be needed to elucidate the molecular mechanisms of ACE2 downregulation. Since the SARS-CoV-2 affects nasal mucosa, GI, the eye, and the nervous system exploring the protective potential of ELVs in other cell types would further expand the scope of our observations beyond the lung. Our results provide a foundation for future research and offer specific mediators for interventions to modify disease risk, progression, and protection of the lung from COVID-19 or other pathologies.

## Methods

### Primary cultures of human alveolar cells and assessment of protein internalization

We have used primary cell cultures of human alveoli, which consist of a mixture of ciliated cells, club cells, type I pneumocytes, and type II pneumocytes (PromoCell, HSAEpC). We have characterized the histology and immunocytochemistry in these primary cultures (Fig. 1a,b,g,h). We performed all the experiments in 48 wells with passage 4 cells seeded at 15,000 cells per cm^2^ density. The cells were incubated to confluency and maintained in the proprietary Medium provided by Promocell with the addition of Pen/Strep and exposed to 0.5 μg of tagged protein per well for 24 h in the presence or absence of 10 ng/ml IL1β (PeProTech Inc., Rocky Hill, NJ Cat# 200-01B) and/or 10 ng/ml TNFα (Cell Sciences Inc., Newburyport MA. Cat# CRH520B). After this period, cells were incubated 10 min with 1/1000 cell mask (Thermo Scientific cat# C37608) medium and fixed using PFA 4%. After fixation, nuclei were stained with 10 μg/ml Hoechst 33342 (Thermo Scientific cat#H3570). To characterize the cell types in culture after fixation, we performed immunocytochemistry using the following primary antibodies: HT1-53, a marker of pneumocytes type I, and HT2-280 (Terrace Biotech cat#HT1-53 and HT2-280); Foxj1 (Santa Cruz Biotech, sc-53139) and β-Tubulin IV marker of pneumocytes type II (Abcam cat# ab179509). ACE2 (Santa Cruz Biotech, sc-390851) and TMPRSS2 (Abcam cat #Ab109131) were used for Immunostain the two mentioned proteins in cell culture.

### ACE2 protein abundance using Jess technology

The western assay was performed using a Jess Protein Simple system (San Jose, CA, USA) following the manufacturer’s protocol. Briefly, samples were lysed with RIPA buffer containing a protease inhibitor cocktail (Sigma, Cat. P8340). Soluble protein concentration was determined by BCA assay (Thermo Fisher Scientific, Cat. 23225) and 0.4 µg used/reaction. Samples were heated at 95 °C/5 min, and 3 µL of each sample were loaded. The 12–230 kDa cartridge (Protein Simple—#SM-W004) was used. Primary antibodies were diluted in antibody diluent 2 buffer (Protein Simple, #042-203), and the working solution of secondary antibodies was provided by the company (Protein Simple, #042-206). For data analysis, the area of spectra that matched the molecular weight of the target protein was used (Fig. 1j). We used the anti-ACE2 antibody from Abcam (cat# ab108252) in a concentration of 1 μg/ml. The standardization was performed using total protein stain and using an anti-GAPDH antibody from Santa Cruz Biotech (cat# Sc-25778).

### Quantification of RNF-146

Western blot was performed from human primary alveolar cells, as described in Calandria et al., 2015 (18). Briefly, cell lysates were produced using RIPA buffer supplemented with protease inhibitor cocktail (Sigma, cat# P8340.St Louis MO). Total protein (30 mg) was mixed with Laemmli buffer containing DTT and loaded in Novex 4–12% precast gels and ran in X-Cell running system at 120 V for 1.5 h. The transference was performed using the Trans Blot Turbo dry transferring system (Bio-Rad, Hercules CA) on low fluorescent background PVDF membranes (GE Healthcare, Piscataway NJ). Membranes were incubated with the corresponding primary antibodies overnight. Primary antibodies used RNF-146 (UC Davis/NIH-Neuromab Lab Facility, cat #75-233) and GAPDH (Satnta Cruz Biotech Cat# sc-47724). After this period, the membranes were incubated with fluorescent-tagged secondary antibodies (GE healthcare, cat# PA45011) for 1 h and imaged. Data was acquired using ChemiDoc MP (Biorad). Densitometric analysis was performed using ImageLab 6.0.1. (Biorad).

### Preparation of tagged RBD and N nucleocapside proteins

We have obtained the Recombinant SARS-CoV-2, S1 Subunit Protein (RBD), and Recombinant SARS-CoV-2 Nucleocapsid Protein from Raybiotech (Peachtree Corners GA, cat# 230-30162-1000 and 230-30164-500 correspondingly). The proteins were labeled using Alexa Fluor™ 546 Protein Labeling Kit from Thermo Scientific (cat# A10237) following manufacturer directions except for the RBD that was purified from the dye with Amicon-Ultra 10K cutoff filters (Merck, Millipore cat#UFC201024) instead of the column provided by the kit has a restrictive MW of 50KD (the recombinant RBD protein was 25KDa). The recovery of the protein and labeling efficiency was measured using nanodrop and was about 80% recovery and 0.02 dye molecules per aminoacid. We added 0.5 μg per well of protein.

### Quantitation of cell surface binding and internalization of tag RBD of the viral S protein

0.5 μg of Alexa 594-conjugated RBD domain belonging to the SARS-CoV-2 virus Spike protein (Raybiotech. Cat. 230-30162-1000) was incubated with human alveolar cells for 24 h. After this period, Cell Mask (Thermofisher Scientific, cat#C37608) was added to a final concentration of 1 in 1000 and left in the incubator for 10 min. The cells were then fixed with 4% Paraformaldehyde in PBS, washed three times, and Nucblue (Thermofisher Scientific cat# R37606) was added for nuclear staining. The images were taken using an Olympus FluoView 3000 laser confocal microscope as z-stacks with a fixed step size of 1.6 μm. The cero was registered for each well specifying − 30 and 30 μm as the lower and upper limit in the Z-axes. Using the Z-drift compensation system, 9 blind positions were set up per well, and the image acquisition was performed automatically. The pictures were processed using Imaris 9.5.1 software (Bitplane) to render the 3D image and assess the position of the different surfaces (elements rendered with the surface function) along the Z-axes. The portion of the tagged protein that was internalized was calculated from the total sum of intensity that crossed the membrane inner limit to the intracellular space in the Z-axes. The proportion was calculated in percentage of internalization.

### Real-time PCR using Taqman probes and SYBR green assay

cDNA was produced using 1 μg of total RNA extracted by RNAeasy (Qiagen, Hilden Germany, cat# 74104). The first strand of cDNA was produced using iScript™ Reverse Transcription Supermix for RT-qPCR (BioRad cat # 1708840). The quantification of Sirt 1, RNF-146, Bcl2, BcL-xl was performed using SYBRgreen assay with primers designed in house (Table 1) using SsoAdvanced Universal SYBR Green Supermix (Biorad cat#1725270). The quantification of ACE2 and TMPRSS2 mRNA was performed using Taqman probes (Biorad cat# qHsaCEP0051563 & qHsaCIP0028919 respectively) labeled with FAM and standardized using PGK1 probe labeled with HEX (Biorad cat# qHsaCEP0050174).

**Table 1:** Primer sequences used to measure mRNA content in human primary lung culture by SYBR green Real-Time Polymerase Chain Reaction.

Gene name	Accession number	Primer	Seq 5′ to 3′
Sirtuin 1	NM_012238.5	SIRT1F	TGCTGGCCTAATAGAGTGGCA
		SIRT1R	CTCAGCGCCATGGAAAATGT
Prohibitin 1	NM_001281496.**1**	PHB1F	AAGGCTCGTTTCTGGGCATCTC
		PHB1R	CTGGACCCTCTCACACGCA
Bcl2l1/BcL-XL	Z23115.1	Bcl-XLF	AGGCGGATTTGAATCTCTTTCTCT
		Bcl-XLR	CCCGGTTGCTCTGAGACATT
RNF-146/Iduna	NM_001242844.1	RNF146F	ATTCCCGAGGATTTCCTTGACA
		RNF146R	GCTCATCGTACTGCCACCA
Bcl2	NM_000633.3	Bcl2F	GATGTGATGCCTCTGCGAAG
		Bcl2R	CATGCTGATGTCTCTGGAATCT

### Infection of human alveolar cells with SARS-CoV-2

The human primary alveolar cells were pre-incubated with 1 uM of ELV-32 Me, ELV-34 Na, ELV-32 AC, ELV-34 AC or their precursors VLC-PUFA 32:6 or 34:6 for 24 h before infection. To infect primary culture cells in chamber slides, a stock of SARS-CoV-2 (USA-WA1/2020 isolate, accession MN985325) was diluted to the appropriate concentration using serum-free DMEM and overlaid on the cells. Briefly, 10,000 TCID50 of virus was added at a volume of 100ul per well and allowed to incubate for 1 h at 37C, with gentle rocking every 15 min. After one hour, the inoculum was removed and replaced with media containing a fresh batch of the indicated lipids. Slides were returned to the incubator and monitored for 3 days. After this period, the media was removed and replaced with 2% paraformaldehyde and left overnight at room temperature to fix the cells and inactivate the virus, facilitating the transfer of the slides from BSL3 to BSL2. The next day, the PFA was replaced with PBS, and the cells were immunostained using antibody anti-Spike protein from Abcam (cat#ab273433), counterstained, and imaged as described above.

### eSight assay

To measure the effects of the lipids on SARS-CoV-2 infection, we used real-time cell analysis (RTCA) to measure impedance with the Agilent eSight system. The day before the assay was initiated, the alveolar cells were at confluence and exposed to 1 uM of ELV-32 Me, ELV-34 Na, ELV-32 AC, ELV-34 AC or their precursors, VLC-PUFA 32:6 or 34:6, followed by incubation in Agilent e-plates. The virus was incubated with the cells at 37C for 1 h. After that, the virus was removed, and the medium was replaced by lipid-containing media. The plates were loaded on the eSight instrument. Impedance was measured every 15 min, and an image was taken every hour for 125 total hrs. The data was analyzed using Agilent RTCA Pro software.

## Supplementary Information

Supplementary Information 1.

Supplementary Information 2.

Supplementary Video 1.

Supplementary Video 2.
