# Supplementary material for: Elovanoids downregulate SARS-CoV-2 cell-entry, canonical mediators and enhance protective signaling in human alveolar cells
Source: Sci Rep. 2021 Jun 10;11:12324. doi: 10.1038/s41598-021-91794-z (PMC8192580; doi:10.1038/s41598-021-91794-z)
Supplement: Supplementary file 1 — Supplementary Information 1. [file 41598_2021_91794_MOESM1_ESM.pdf]

Elovanoids downregulate SARS-CoV-2 cell-entry, canonical mediators and enhance protective signaling in human alveolar cells

Jorgelina M. Calandria[1], Surjyadipta Bhattacharjee[1], Nicholas J. Maness[2,3], Marie-Audrey I. Kautzmann[1], Aram Asatryan[1], William C. Gordon[1], Khanh V. Do[1], Bokkyoo Jun[1], Pranab K. Mukherjee[1], Nicos A. Petasis[4], and Nicolas G. Bazan[1]*

[1]Neuroscience Center of Excellence, School of Medicine, Louisiana State University Health New Orleans, 2020 Gravier Street, Suite D, New Orleans, USA.

[2]Tulane National Primate Research Center, Covington, LA, USA.

[3]Department of Microbiology and Immunology, Tulane University School of Medicine, New Orleans, LA, USA.

[4]Department of Chemistry and Loker Hydrocarbon Research Institute, University of Southern California, Los Angeles, CA, USA.

*Correspondence to: Nicolas G. Bazan, Neuroscience Center of Excellence, School of Medicine, Louisiana State University Health New Orleans, 2020 Gravier Street, Suite D, New Orleans, LA, 70112, USA; Phone: 504-599-0832; E-mail: nbazan@lsuhsc.edu.

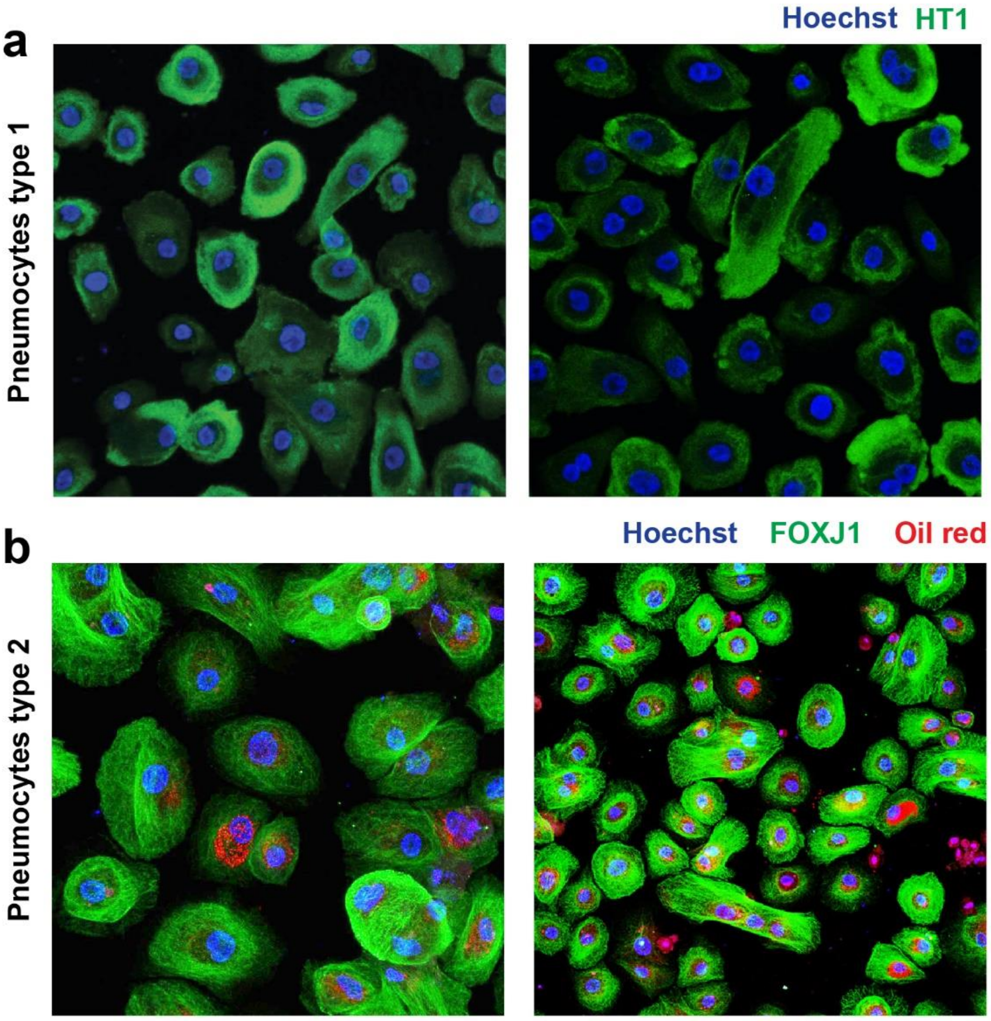

Extended Data Fig. 1. Pneumocyte markers in chamber slide cultures. Type I pneumocytes labeled (green) with the specific marker HT1-53 (upper panels). Type II lung pneumocytes labeled with a ciliated cell marker Foxj1 (green), which is required for cilia formation and is an early marker of epithelial cell differentiation, recovery, and function, and Oil Red O (red), a marker for the type II lipid/lamellar bodies (bottom panels). Nuclei were labeled with Hoechst (blue).

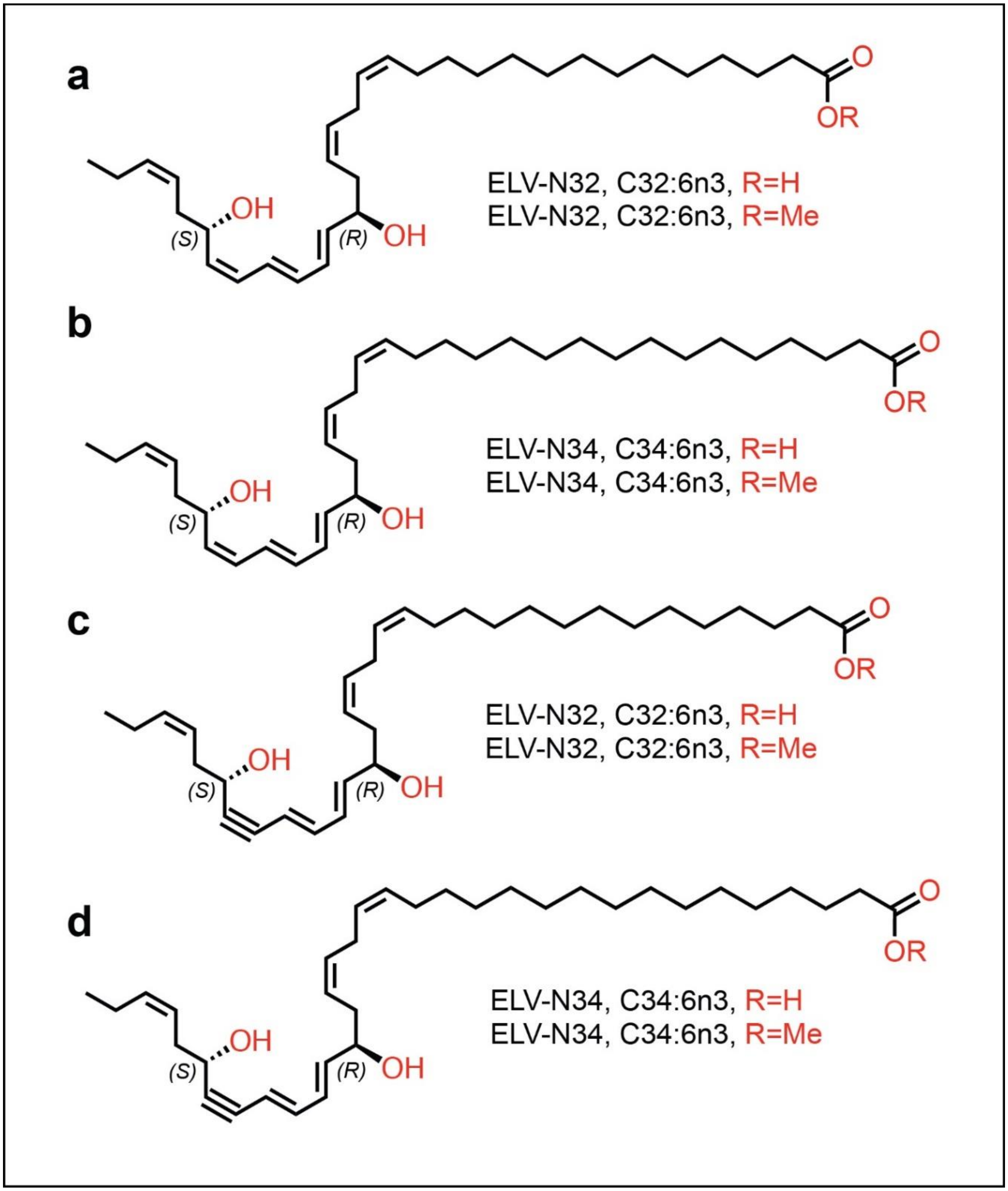

Extended Data Fig. 2. Chemical structures of the elovanoids N32 (top) and N34 (second). ELV-N32, R denotes the substituent in the carbon located is H = hydrogen or Me = methyl-ester. The third and fourth molecule from top to bottom shows the triple bond that make the molecules acetylenic.

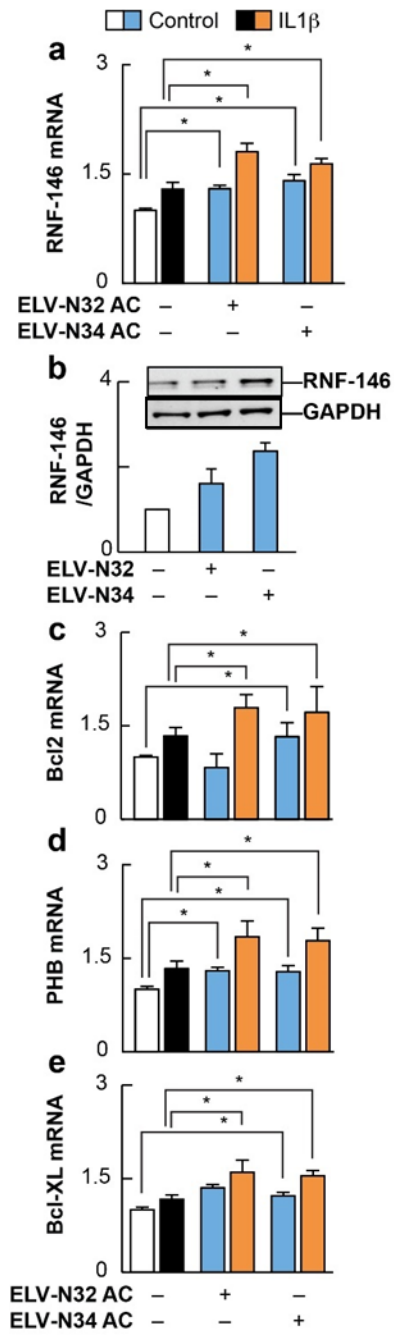

Extended Data Fig. 3. a,c,d,e. Semi-quantitave real time PCR quantitation of target of elovanoids N32 and N34, RNF-146 (alias Iduna), Prohibitin (PHB), Bcl2 and Bcl-XL (Primers in Table 1), in alveolar cells exposed to the acetylene form of the ELVs for 24 hours in the presence or absence of 10ng/ml IL1β. **b,** Western blot assay on alveolar cells exposed for 24h to Elovanoids N32 and N34 with the substituent R=methyl ester.

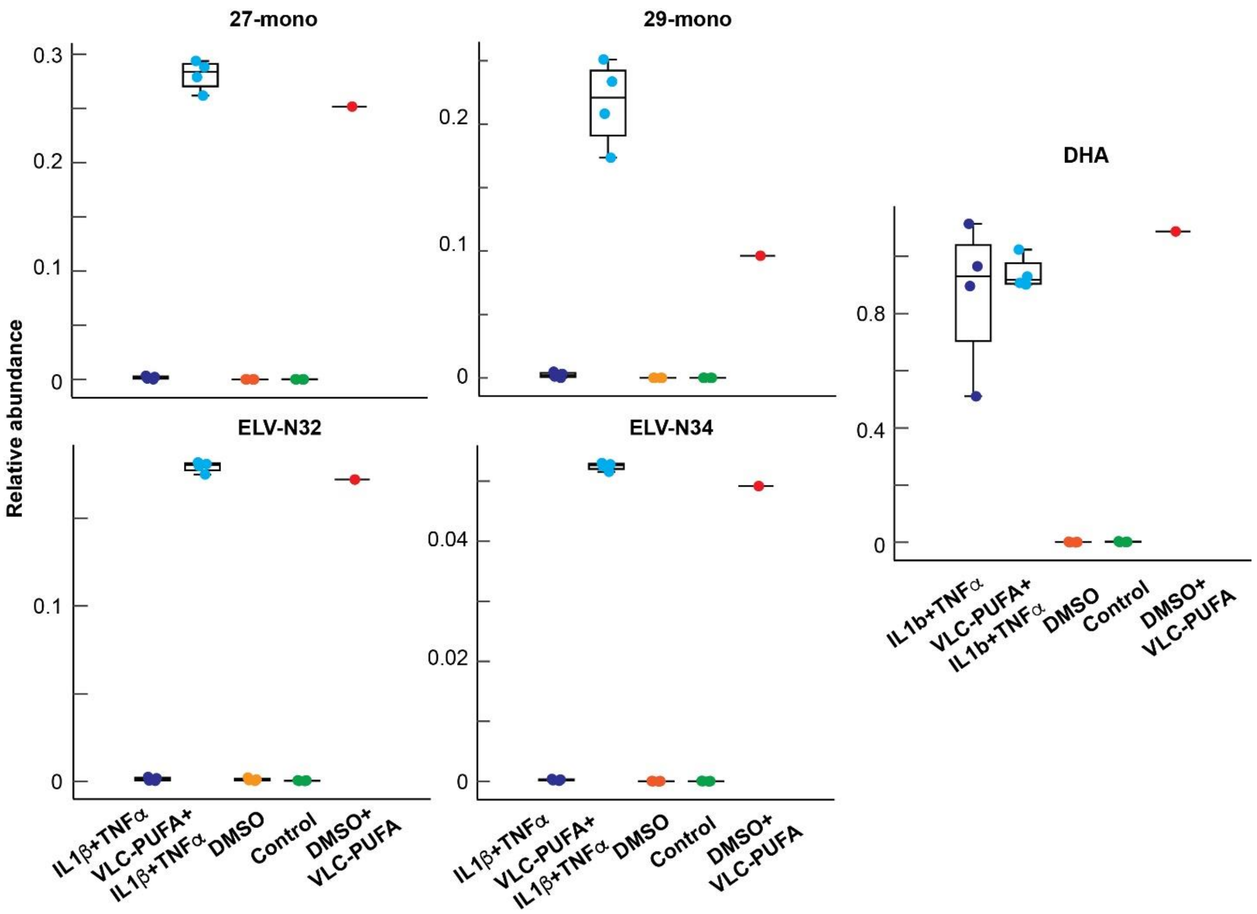

Extended Data Fig. 4. Relative abundance of the elovanoids N32 and N34 (bottom panels) and their intermediates 27 mono-hydroxy and 29 mon-hydroxy (top panels) when the alveolar cells are exposed to the precursors VLC-PUFAs: 32:6 and 34:6 in the presence or absence of 10ng/ml IL1β and 10 ng/ml TNFα for 24 hours. DHA abundance was analyzed as a specificity control. The plot shows the box upper limit 3rd Quartile, bottom side 1st quartile, middle line: the median and the whiskers denote the maximum and minimum observations. *p<0.05 in t-test comparisons with the respective control. Repetition of the experiment of Fig. 2 in 24 well plates.

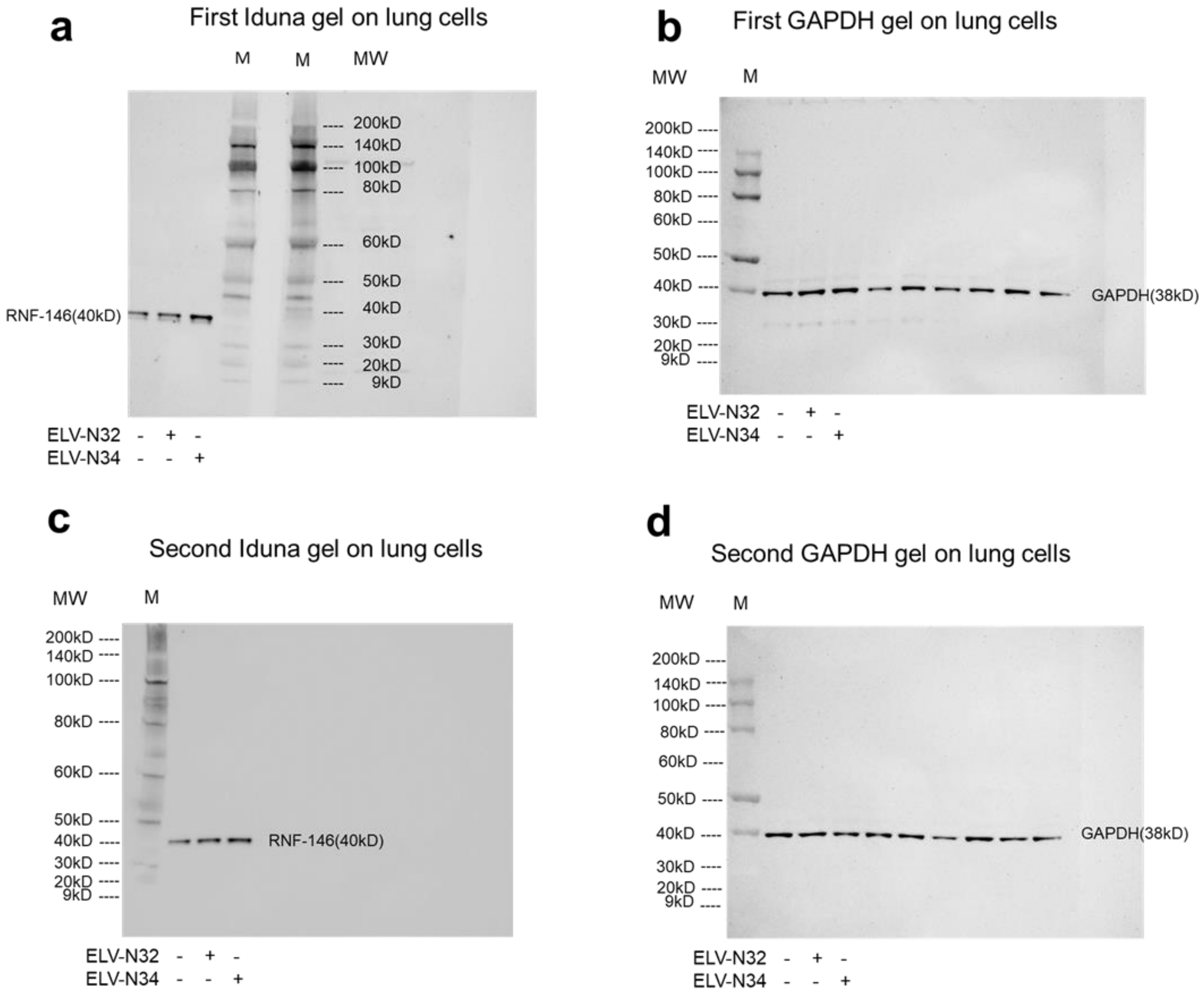

Extended Data Fig. 5. a-d. Western blot membranes shown in Extended Data Fig. 3b. Entire membrane probed with anti-RNF46 antibody (**a,c**; duplicates) and ran in another gel to probe with anti-GADPH (**b,d**; duplicates). The GAPDH was run in another gel because the bands for both proteins overlap due to their similar molecular weight. The ladder used was Biotinylated Protein Ladder (Cell signaling #7727). Samples used in the quantification in Extended Data Fig. 3b; first three lanes of **a**, and first three lanes after the marker of **b-d**. The remaining lanes are other irrelevant samples.

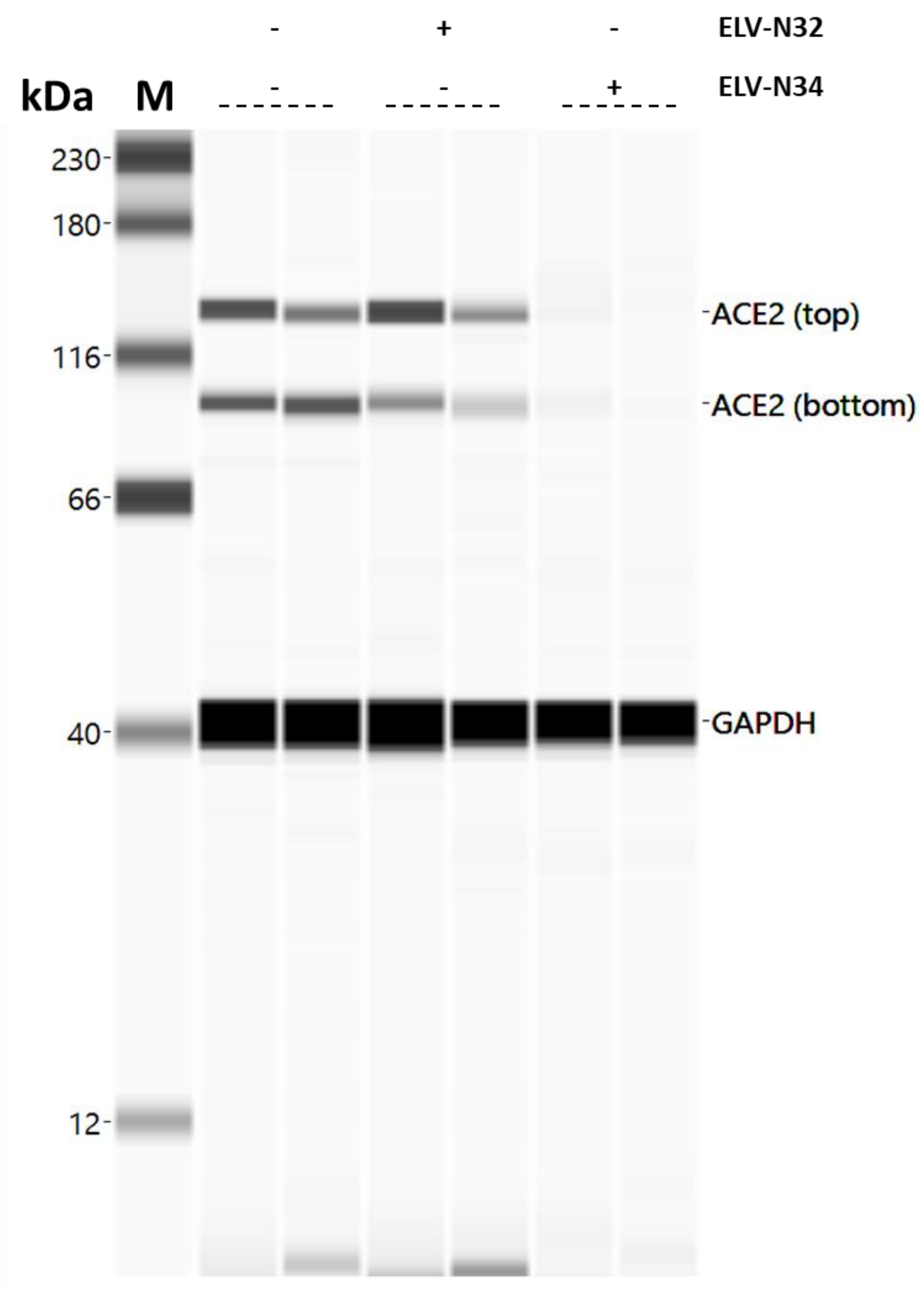

Extended Data Fig. 6. Jess capillary blots shown in Fig. 1i. Entire capillary blot probed with anti-ACE2 antibody (**a**) showing upper and lower bands, ladder and GAPDH bands. The integration of the area under the peak of intensity for the lower band values was computed into the results shown in Fig. 1i.

Extended Data Video 1. Time frame composition of bright-field images taken every hour on the same spot showing the behavior of the cells exposed to 1 μM Elovanoid 32 Me and 1 μM 32:6. The AVI extension movie shows that the lipids have no toxic effects on the lung cells. The impedance shows no change during the five days of continuous recording. The images and the impedance were taken in an XCelligence RTCA Analyzer (Agilent, Santa Clara, CA). The AVI extension (2 images per second) was assembled in ImageJ NIH (imagej.nih.gov/ij/list.html).

Extended Data Video 2. Time frame composition of bright-field images taken every hour on the same spot showing the behavior of the cells infected with 1000 TCID50 of SARS-CoV-2. The AVI extension (2 images per second) was assembled in ImageJ-NIH (imagej.nih.gov/ij/list.html). The inferior left quadrant shows infected cells lift from the well around 29 to 30 hours (images) after another cluster of cells (debris) is placed on top. The space left behind is rapidly covered by the neighbor cells, so the impedance is unchanged. The cluster emerging from the affected cells spreads little clusters in the subsequent hours. The images and the impedance were taken in an XCelligence RTCA Analyzer (Agilent, Santa Clara, CA).
