# Supplementary figures and images for: Elovanoids downregulate SARS-CoV-2 cell-entry, canonical mediators and enhance protective signaling in human alveolar cells
Source: Sci Rep. 2021 Jun 10;11:12324. doi: 10.1038/s41598-021-91794-z (PMC8192580; doi:10.1038/s41598-021-91794-z)

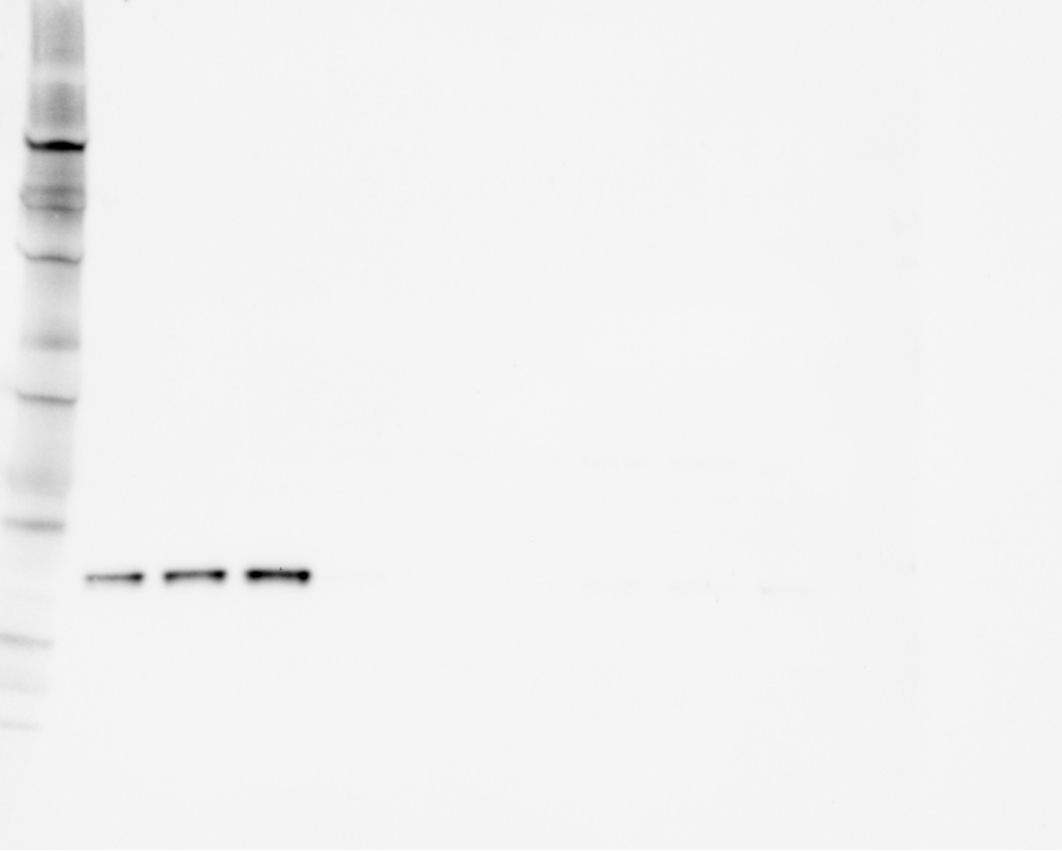

Supplement: Supplementary file 2 — Supplementary Information 2. [file 41598_2021_91794_MOESM2_ESM.zip › pranab 2020-07-15 14h31m15s(Chemiluminescence) 5c low exposed.jpg]

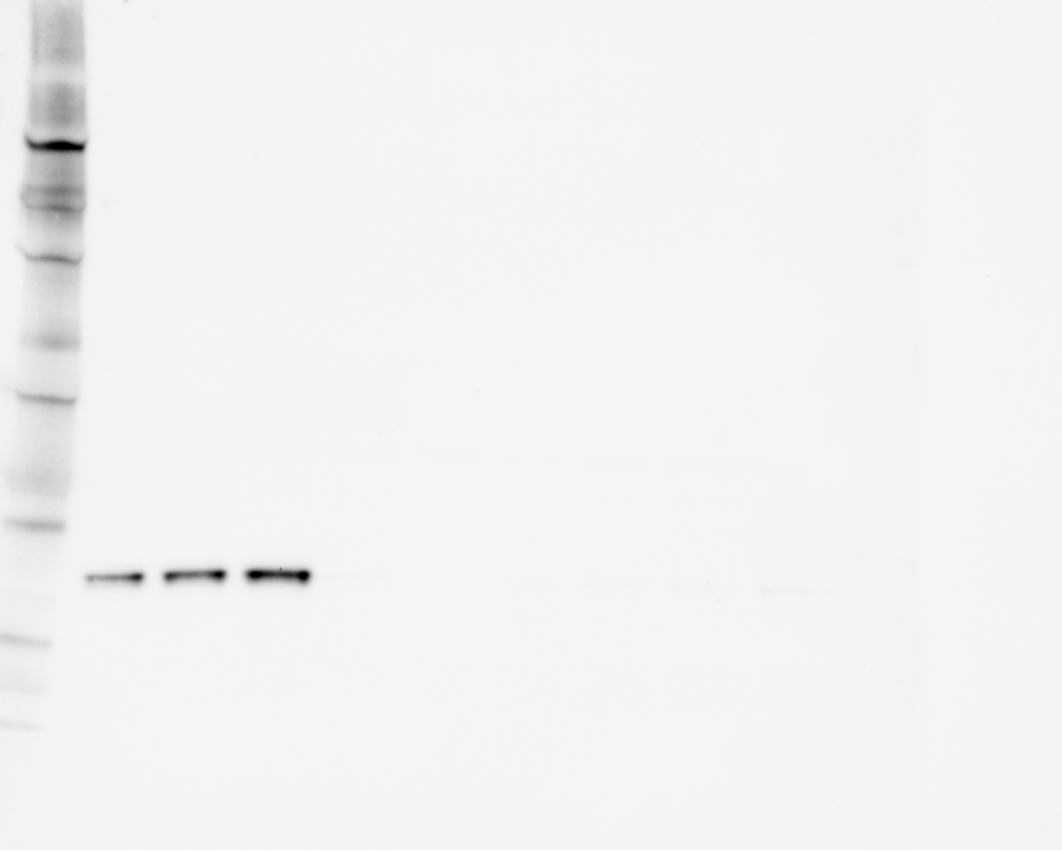

Supplement: Supplementary file 2 — Supplementary Information 2. [file 41598_2021_91794_MOESM2_ESM.zip › pranab 2020-07-15 14h31m15s(Chemiluminescence) 5c low exposed.tif]

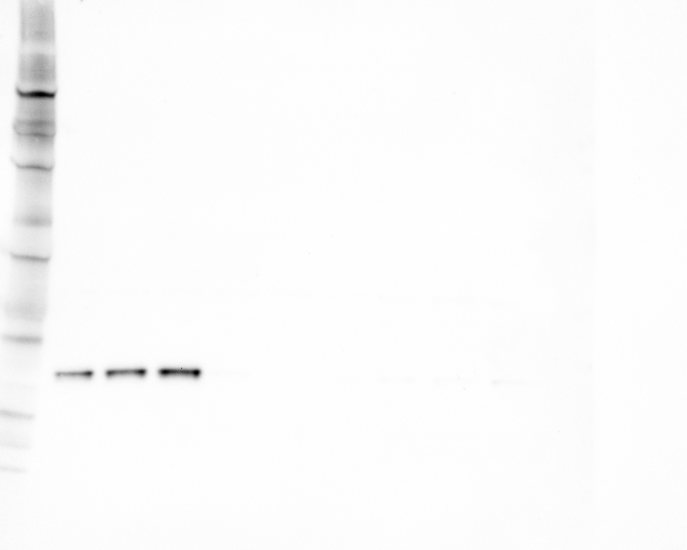

Supplement: Supplementary file 2 — Supplementary Information 2. [file 41598_2021_91794_MOESM2_ESM.zip › pranab 2020-07-15 14h31m15s(Chemiluminescence).raw16 5c low exposed.tif]

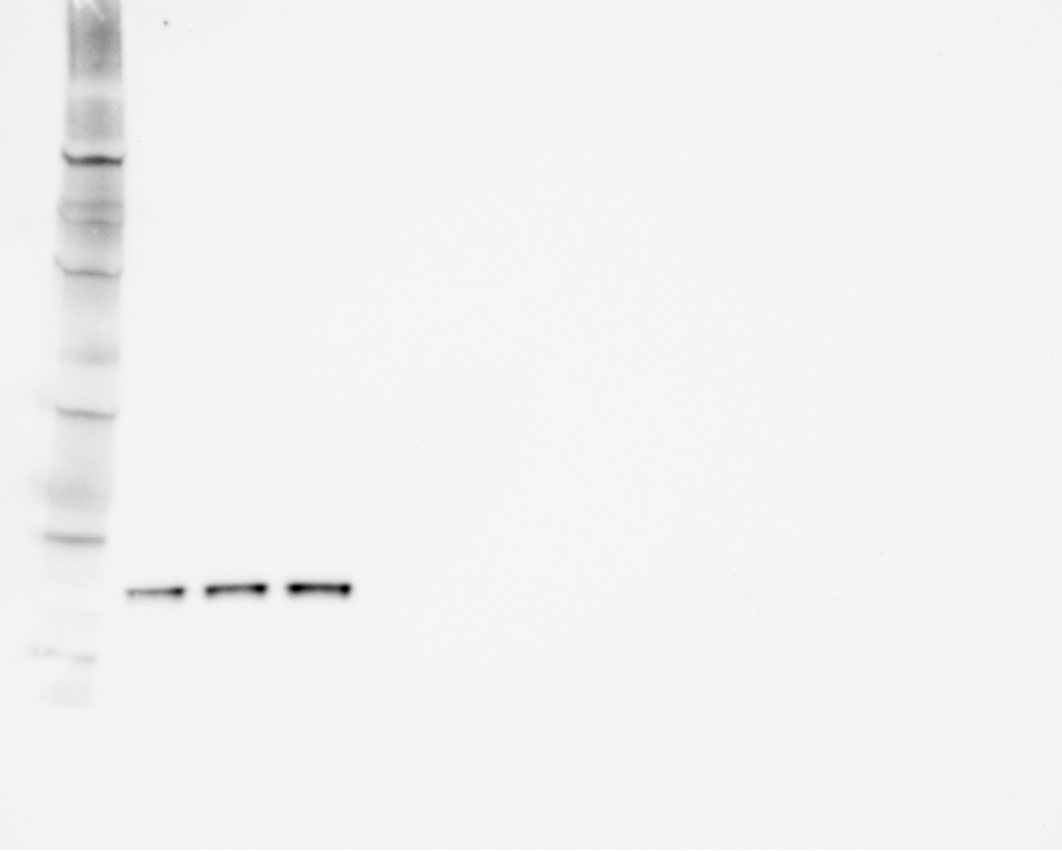

Supplement: Supplementary file 2 — Supplementary Information 2. [file 41598_2021_91794_MOESM2_ESM.zip › pranab 2020-07-15 14h49m25s(Chemiluminescence) 5c extended data.jpg]

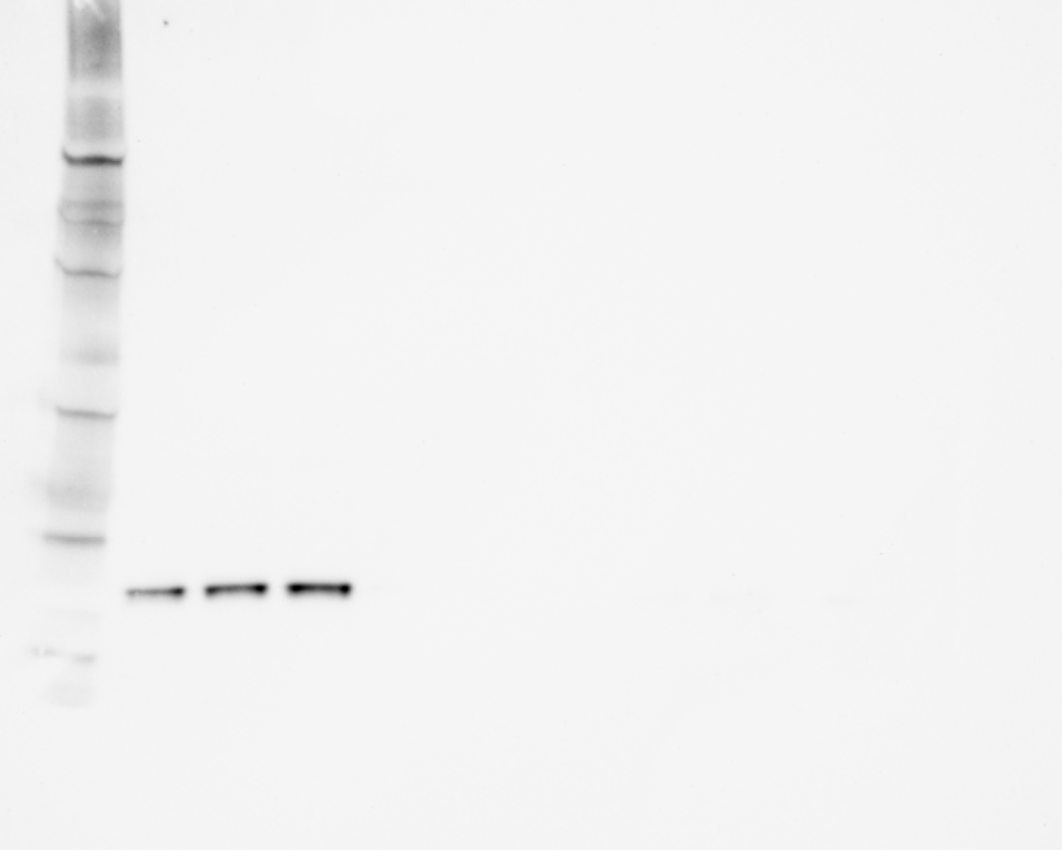

Supplement: Supplementary file 2 — Supplementary Information 2. [file 41598_2021_91794_MOESM2_ESM.zip › pranab 2020-07-15 14h49m25s(Chemiluminescence) 5c extended data.tif]

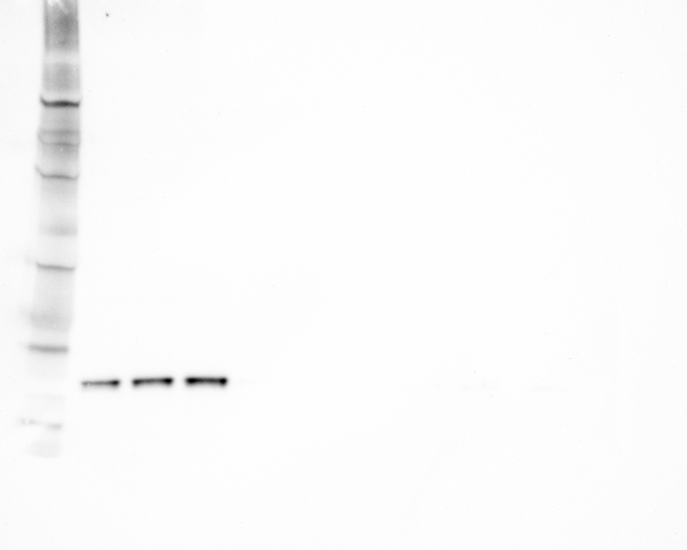

Supplement: Supplementary file 2 — Supplementary Information 2. [file 41598_2021_91794_MOESM2_ESM.zip › pranab 2020-07-15 14h49m25s(Chemiluminescence).raw16 5c extended data.tif]

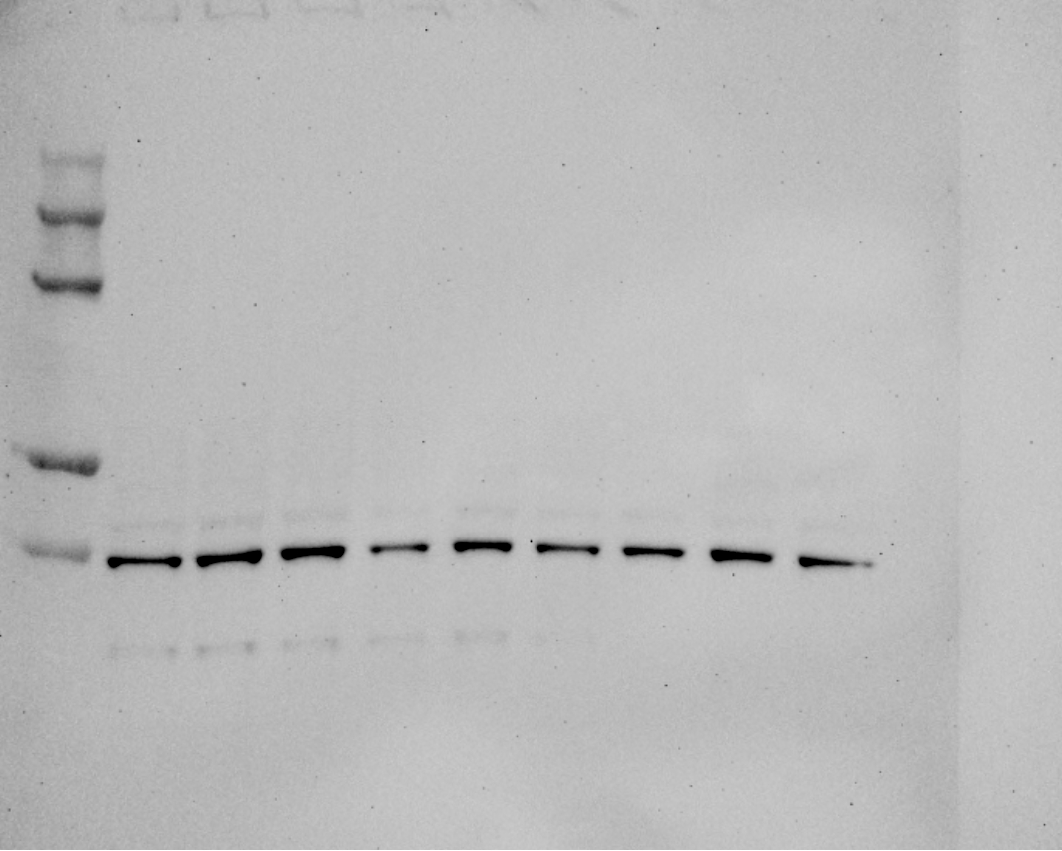

Supplement: Supplementary file 2 — Supplementary Information 2. [file 41598_2021_91794_MOESM2_ESM.zip › pranab 2020-07-16 16h38m57s(Chemiluminescence) 5b high exposed.jpg]

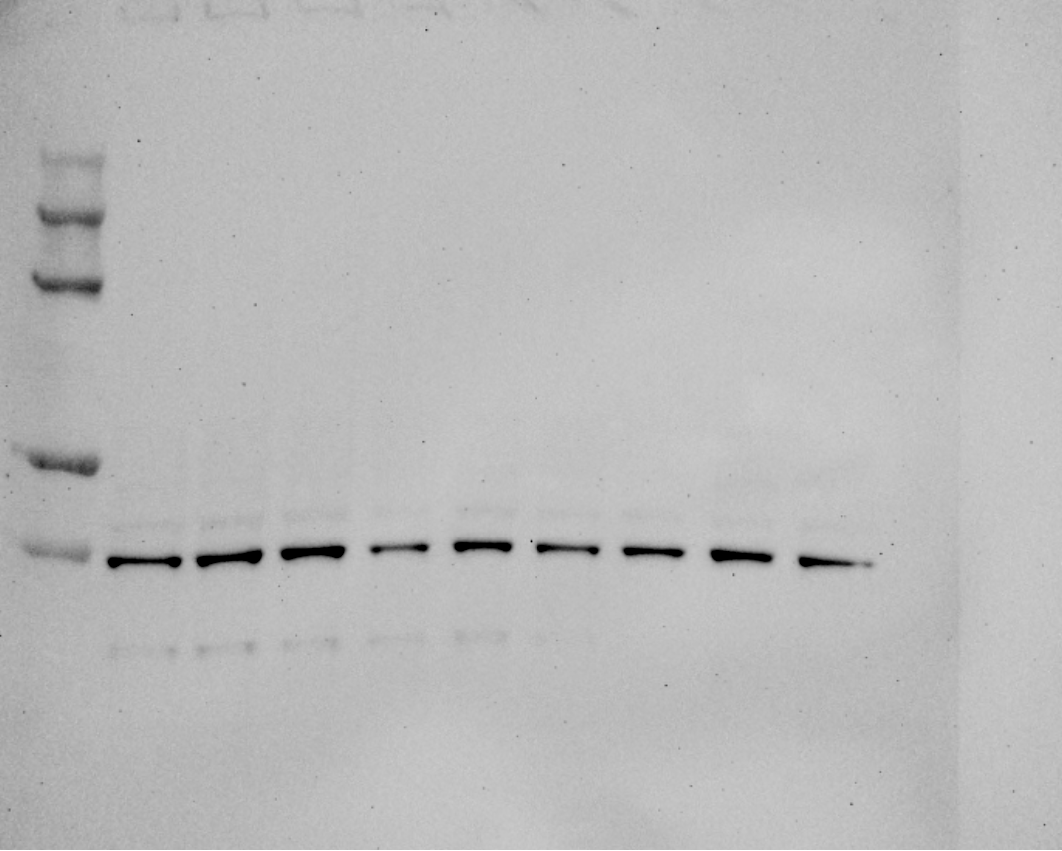

Supplement: Supplementary file 2 — Supplementary Information 2. [file 41598_2021_91794_MOESM2_ESM.zip › pranab 2020-07-16 16h38m57s(Chemiluminescence) 5b high exposed.tif]

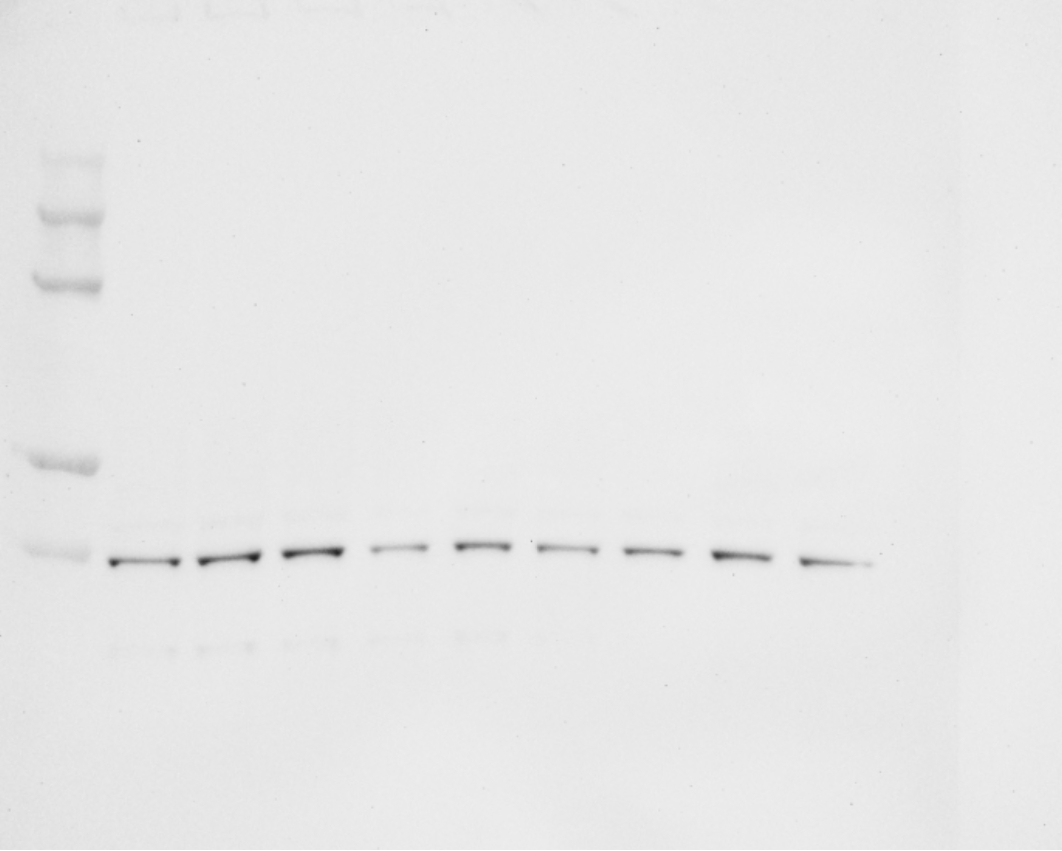

Supplement: Supplementary file 2 — Supplementary Information 2. [file 41598_2021_91794_MOESM2_ESM.zip › pranab 2020-07-16 16h38m57s(Chemiluminescence) 5b intermediate exposed.jpg]

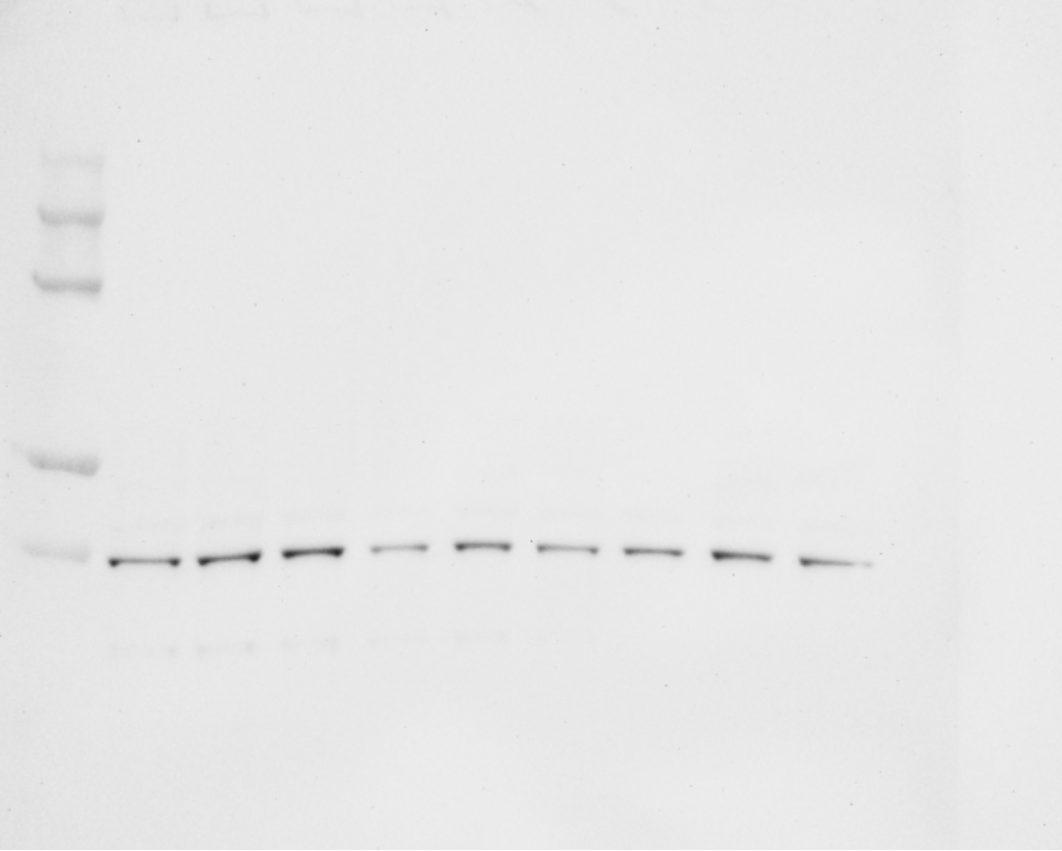

Supplement: Supplementary file 2 — Supplementary Information 2. [file 41598_2021_91794_MOESM2_ESM.zip › pranab 2020-07-16 16h38m57s(Chemiluminescence) 5b intermediate exposed.tif]

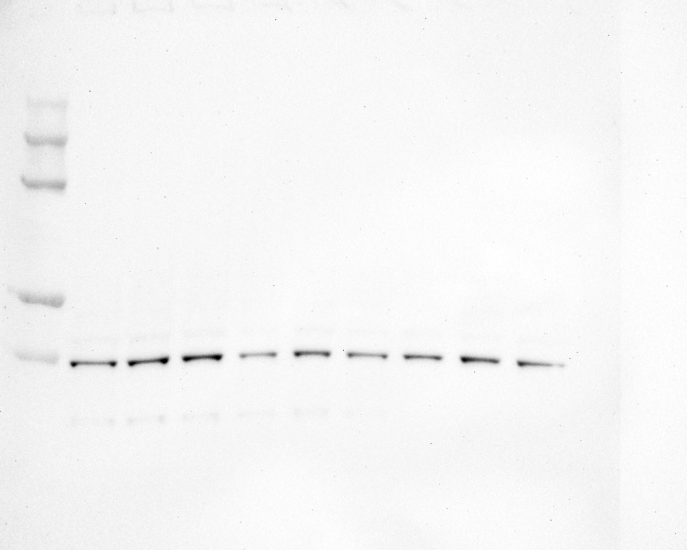

Supplement: Supplementary file 2 — Supplementary Information 2. [file 41598_2021_91794_MOESM2_ESM.zip › pranab 2020-07-16 16h38m57s(Chemiluminescence).raw16 5b intermediate exposed.tif]

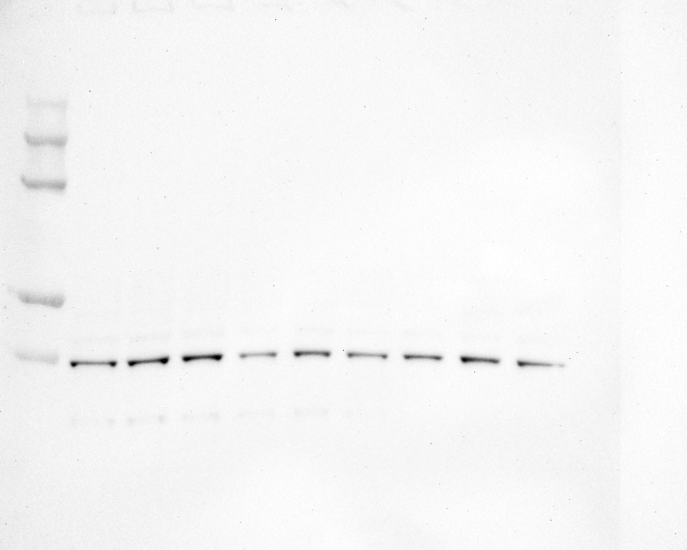

Supplement: Supplementary file 2 — Supplementary Information 2. [file 41598_2021_91794_MOESM2_ESM.zip › pranab 2020-07-16 16h38m57s(Chemiluminescence).raw16 5b low exposed.tif]

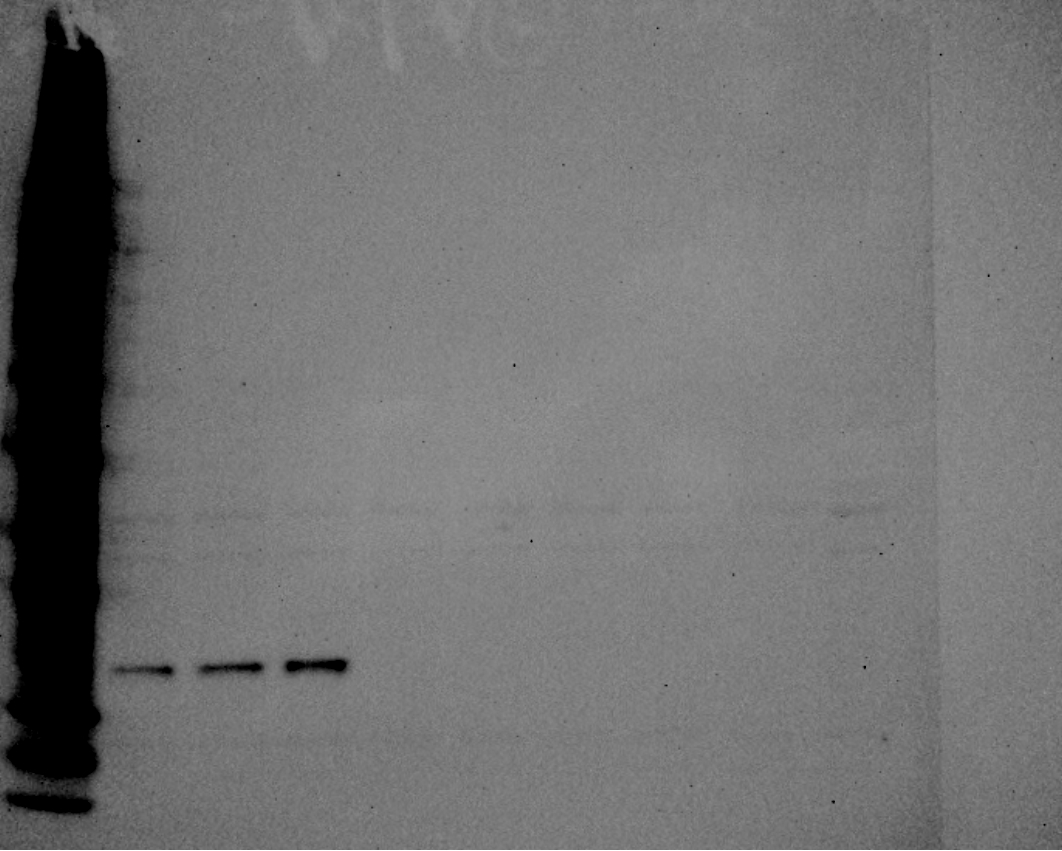

Supplement: Supplementary file 2 — Supplementary Information 2. [file 41598_2021_91794_MOESM2_ESM.zip › pranab 2020-07-17 12h39m50s(Chemiluminescence) 5c overexposed.jpg]

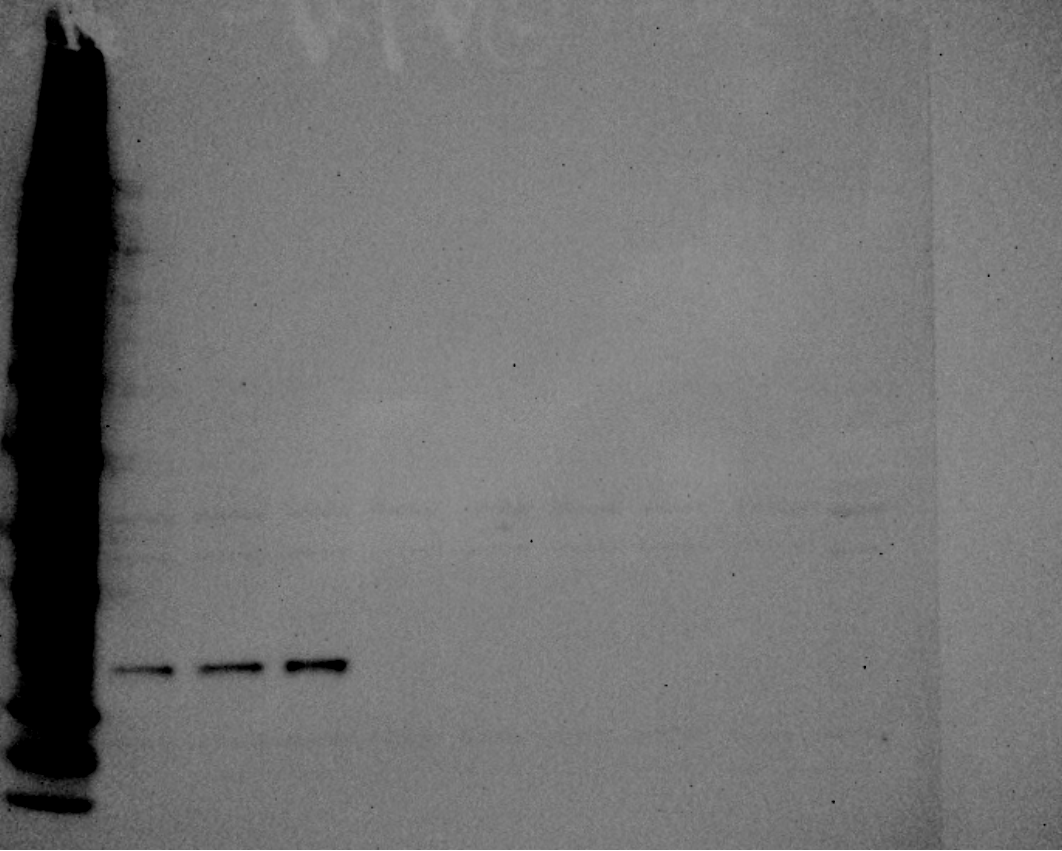

Supplement: Supplementary file 2 — Supplementary Information 2. [file 41598_2021_91794_MOESM2_ESM.zip › pranab 2020-07-17 12h39m50s(Chemiluminescence) 5c overexposed.tif]

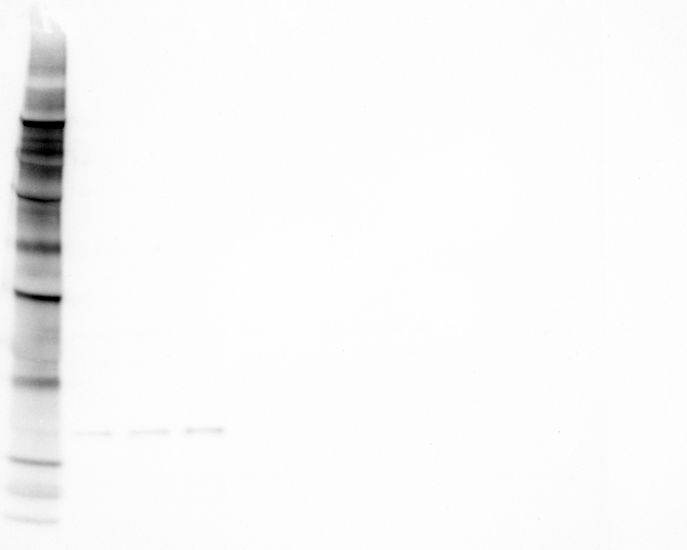

Supplement: Supplementary file 2 — Supplementary Information 2. [file 41598_2021_91794_MOESM2_ESM.zip › pranab 2020-07-17 12h39m50s(Chemiluminescence).raw16 5c overexposed.tif]
